# Combined inhibition of BET proteins and class I HDACs synergistically induces apoptosis in urothelial carcinoma cell lines

**DOI:** 10.1186/s13148-017-0434-3

**Published:** 2018-01-04

**Authors:** Alexander S. Hölscher, Wolfgang A. Schulz, Maria Pinkerneil, Günter Niegisch, Michèle J. Hoffmann

**Affiliations:** Department of Urology, Medical Faculty, Heinrich-Heine-University, Duesseldorf, Moorenstr. 5, 40225 Duesseldorf, Germany

**Keywords:** BET inhibitor, JQ1, HDACi, Romidepsin, Bladder cancer, Apoptosis

## Abstract

**Background:**

New efficient therapies for urothelial carcinoma (UC) are urgently required. Small-molecule drugs targeting chromatin regulators are reasonable candidates because these regulators are frequently mutated or deregulated in UC. Indeed, in previous work, Romidepsin, which targets class I histone deacetylases (HDAC), efficiently killed UC cells, but did not elicit canonical apoptosis and affected benign urothelial cells indiscriminately. Combinations of HDAC inhibitors with JQ1, an inhibitor of bromodomain-containing acetylation reader proteins like BRD4, which promote especially the transcription of pro-tumorigenic genes, have shown efficacy in several tumor types. We therefore investigated the effects of combined Romidepsin and JQ1 treatment on UC and benign urothelial control cells.

**Results:**

JQ1 alone induced cell cycle arrest, but only limited apoptosis in eight UC cell lines with strongly varying IC_50_ values between 0.18 and 10 μM. Comparable effects were achieved by siRNA-mediated knockdown of BRD4. Romidepsin and JQ1 acted in a synergistic manner across all UC cell lines, efficiently inhibiting cell cycle progression, suppressing clonogenic growth, and inducing caspase-dependent apoptosis. Benign control cells were growth-arrested without apoptosis induction, but retained long-term proliferation capacity. In UC cells, anti-apoptotic and oncogenic factors Survivin, BCL-2, BCL-XL, c-MYC, EZH2 and SKP2 were consistently downregulated by the drug combination and AKT phosphorylation was diminished. Around the transcriptional start sites of these genes, the drug combination enhanced H3K27 acetylation, but decreased H3K4 trimethylation. The cell cycle inhibitor CDKN1C/p57^KIP2^ was dramatically induced at mRNA and protein levels. However, Cas9-mediated CDKN1C/p57^KIP2^ knockout did not rescue UC cells from apoptosis.

**Conclusion:**

Our results demonstrate significant synergistic effects on induction of apoptosis in UC cells by the combination treatment with JQ1 and Romidepsin, but only minor effects in benign cells. Thus, this study established a promising new small-molecule combination therapy approach for UC.

**Electronic supplementary material:**

The online version of this article (10.1186/s13148-017-0434-3) contains supplementary material, which is available to authorized users.

## Background

For more than 30 years, chemotherapy of invasive urothelial carcinoma has been based on combinations of cisplatin with other cytotoxic drugs. This treatment is moderately efficacious and limited by frequent development of resistance and toxicity in the often elderly patients. Novel drugs targeting growth factor receptors or signal transduction pathways have so far not yielded significant benefits in clinical trials and have therefore not been introduced into clinical practice. Intriguingly, among all cancer types, urothelial carcinoma appears to have the highest prevalence of mutations in chromatin regulator proteins, including various components of the trithorax-like histone-modifying and SWI/SNF1 chromatin remodeling protein complexes [1]. It is therefore reasonable to assume that epigenetic inhibitors represent an alternative approach to chemotherapy of urothelial carcinoma.

Many epigenetic inhibitors target the activity of enzymes modifying histones or DNA. For instance, HDAC inhibitors (HDACi) interfere with the enzymatic activity of histone deacetylases and are considered good drug candidates. Our previous comprehensive analysis of expression of different HDACs isoenzymes in UC and their suitability as therapeutic targets revealed HDAC class I enzymes as the best targets for HDACi in UC therapy [2]. Drugs targeting specific class I HDACs like Romidepsin, Givinostat, or 4SC-202 most efficiently inhibited cell proliferation and caused cell death in UC cells. Despite their efficacy, these compounds do not seem optimal for treatment of UC on their own, because cell death occurred only partly by apoptosis and proliferation of benign urothelial control cells was also efficiently blocked [3, 4]. We therefore investigated the combination of Romidepsin with the BET inhibitor JQ1, which has been proposed to synergize with HDACi in several cancer types and to induce a canonical apoptotic response [5–8]. In pancreatic adenocarcinoma, the synergism was ascribed to several interacting mechanisms, encompassing inhibition of AKT signaling, increased STAT3 phosphorylation, and prominent induction of the CDK inhibitor p57^KIP2^ [8].

JQ1 is the best characterized representative of a novel class of compounds which blocks binding of chromatin proteins by targeting domains recognizing histone modifications. JQ1 specifically targets the bromodomains of transcriptional coactivators like “bromodomain and extra-terminal” (BET) proteins, especially BRD proteins, and appears to be particularly effective in cancers dependent on MYC transcription factors, which are not well druggable by other means [9]. The best studied BET protein BRD4 has been shown to be overexpressed in UC tissues [10] correlating with grade, progression towards metastatic disease, and poor overall survival [11]. As an epigenetic reader of acetylation marks at histone tails, BRD4 functions as a scaffold protein linking chromatin remodeling and transcriptional regulation to cell cycle progression. A newly discovered histone acetyltransferase activity for H3K122 further contributes to chromatin decompaction and transcription activation [12]. Inhibition of BRD4 in particular disrupts super enhancers and represses the oncogenes *c-MYC* and *EZH2* [13, 14]. A pioneer study by Wu et al. on BRD4 in UC revealed its upregulation in cancer tissues and inhibition of cell proliferation by JQ1 in two related UC cell lines, T24 and EJ [10]. Knockdown of *BRD4* likewise inhibited proliferation of these UC cell lines. The authors ascribe these effects to inhibition of *c-MYC* and subsequent downregulation of *EZH2*.

In the present study, we therefore investigated whether JQ1 exerts antineoplastic effects on a broader range of UC cell lines which cover the heterogeneity of urothelial carcinoma more comprehensively. Indeed, its efficacy varied between the cell lines, and JQ1 neither suppressed clonogenic growth nor elicited pronounced apoptosis consistently. The combination of JQ1 with Romidepsin however displayed strong synergies in tumor cell growth suppression and apoptosis induction across all cell lines; concomitantly, histone acetylation was broadly enhanced. As in pancreatic adenocarcinoma cells, p57^KIP2^ emerged as one factor synergistically responding to the combination treatment. Surprisingly, however, p57^KIP2^ knockout rather enhanced apoptosis in UC cells.

## Methods

### Cell culture, transfections, drug exposure

BRD4 expression, effects of siRNA-mediated knockdown, and drug exposure were studied in a range of urothelial carcinoma cell lines (UCCs) representing the heterogeneity of urothelial carcinoma, namely, VM-Cub1, RT-112, T24, 5637, UM-UC-3, HT-1376, 639-V, BFTC-905, J-82, and SW-1710. All cell lines were regularly authenticated by STR profiling and checked for mycoplasm contamination. As normal cell controls, we used the normal urothelial cell lines HBLAK [15] and TERT-NHUC, a culture of primary urothelial cells (NHUC) [16], and benign immortalized fetal kidney HEK-293 cells.

UCCs and HEK-293 cells were cultured in DMEM GlutaMAX-I (Gibco, Darmstadt, Germany) supplemented with 4.5 g/l d-glucose, pyruvate, and 10% FBS (Biochrom, Berlin, Germany). HBLAK cells were cultured in CnT-Prime Epithelial Culture Medium (CELLnTEC, Bern, Switzerland). TERT-NHUC cells were cultured in keratinocyte serum-free medium (Gibco) supplemented with 0.35 μg/ml *N*-epinephrine and 0.33 mg/ml hydrocortisone. NHUC were cultured in keratinocyte serum-free medium (Gibco) supplemented with penicillin/streptomycin, EGF, and BPE. Primary urothelial carcinoma cultures were established from fresh transurethral resectates and cultured in Epilife Medium (Gibco) supplemented with 0.5 ng/ml EGF, 25 μg/ml BPE, 1% nonessential amino acids (Invitrogen, Darmstadt, Germany), 1% ITS mix (Invitrogen), 3 mM glycine, and 10% fibroblast-conditioned medium on a collagen IV matrix [17]. All cells were cultured at 37 °C and 5% CO_2_.

For siRNA-mediated knockdown, cells cultured in six-well plates were transfected with 8 nM BRD4 ON-TARGET plus BRD4 siRNA-SMART pool (L-004937-00-0005, Dharmacon, Freiburg, Germany) or ON-Target plus Control pool (D-001810-10-05, Dharmacon) using Lipofectamine RNAiMAX (Life Technologies, Darmstadt, Germany) and assayed 48, 72, or 120 h post-transfection.

For BRD4 overexpression, cells were transfected with p6344 pcDNA4-TO-HA-Brd4FL (Addgene plasmid #31351) [18] or empty vector (pcDNA4-TO) using X-tremeGENE 9 DNA Transfection Reagent (Roche, Penzberg, Germany).

Romidepsin and (+)-JQ1 were purchased from Selleck Chemicals (Munich, Germany) and dissolved in DMSO. Control cells were treated with DMSO only. Drugs were added 24 h after cell seeding. Pan-Caspase inhibitor Q-VD-Oph (SML0063, Sigma Aldrich, Hamburg, Germany) was dissolved in DMSO and used at 30 μmol/l.

Drug concentrations for combination treatments were adjusted for each cell line since IC_50_ values for both compounds varied strongly (Table 1 upper part).

**Table 1:** Treatment doses for single and combined treatment. Eight UCCs, two immortalized benign urothelial cells (TERT-NHUC, HBLAK), primary urothelial cells (NHUC), and HEK-293 cells were treated with JQ1 at a fixed range of concentrations. Cell viability was measured by MTT- assay 72 h later. IC_50_ values for each cell line were determined (upper part). Cell viability results were used according to the Chou-Talalay method to determine doses for the combination treatment with synergistic effects for each investigated cell line. The respective doses were applied for further functional characterization for 48 h (lower part)

Urothelial carcinoma cell lines		Control cell lines	
	JQ1 IC_50_ [μmol/l]		JQ1 IC_50_ [μmol/l]
VM-Cub1	0.18	TERT-NHUC	0.4
RT-112	0.19	HBLAK	0.4
T24	0.23	NHUC	0.26
5637	0.39	HEK-293	0.26
UM-UC-3	2.6		
HT-1376	5.2		
639-V	6.8		
BFTC-905	10		
JQ1 + Romidepsin combination doses			
	JQ1 [μmol/l]		Romidepsin [nmol/l]
Vm-Cub1	0.22		2.2
UM-UC-3	1		2
T24	0.22		2.2
639-V	1.8		1.6
HBLAK	0.4		0.89

### Determination of viability

Viability of cells treated with JQ1 was measured after 72 h by 3-(4,5-dimetylthiazol-2-yl)-2,5-diphenyltetrazolium bromide dye reduction assay (MTT, Sigma Aldrich). Viability of cells after siRNA-mediated BRD4 knockdown or treatment with Q-VD-Oph was measured via total cellular ATP using CellTiter-Glo Assay (Promega, Mannheim, Germany).

### Calculation of IC_50_ values and drug synergy

For determination of IC_50_ values, JQ1 was added in defined concentration ranges. For determination of drug synergy, JQ1 and Romidepsin were used in fixed dose ratios. For each cell line, individual dose ratios were chosen based on the IC_50_ of the individual drugs (Table 1 upper part). For each cell line, at least five different combinations of concentrations were applied and then analyzed by the Chou-Talalay method using CompuSyn software [19]. The final cell line-dependent concentrations for the 48-h combination treatment used for subsequent analyses are given in Table 1 (lower part).

### Colony-forming assay and Giemsa staining

For colony-forming assays, cells were seeded into six-well plates at a density of 1000 cells/well 48 h post-drug treatment and either 48, 72, or 120 h post-siRNA transfection. After 10–15 days, cells were fixed in methanol and stained with Giemsa (Merck, Darmstadt, Germany).

### Flow cytometry

Cell cycle analyses were performed 48 h after treatment with individual drugs or their combinations and 48, 72, and 120 h post-siRNA transfections. Detached cells in supernatant and attached cells were collected and stained with buffer containing 50 μg/ml propidium iodide, 0.1% sodium citrate, and 0.1% Triton X-100 for 1 h at room temperature. To assess apoptotic cell death and necrosis, cells were incubated with Annexin V-FITC (31490013, Immunotools, Friesoythe, Germany), Annexin V binding buffer, and propidium iodide at 2 μg/ml. Flow cytometry was done using the MACSQuant Analyzer (Miltenyi Biotech, Bergisch Gladbach, Germany) and MACSQuantify software as previously described [20].

### RNA isolation, cDNA synthesis, and qRT-PCR

Total mRNA was isolated using the Qiagen RNeasy Mini Kit (Qiagen, Hilden, Germany) according to the manufacturer’s protocol. cDNA synthesis was performed using the QuantiTect Reverse Transcription Kit (Qiagen) with an extended incubation time of 30 min at 42 °C. qRT-PCR was performed using the QuantiTect SYBR Green RT-PCR Kit (Qiagen) and self-designed primers for the target genes and the reference gene *TBP* (TATA-box-binding protein) on the LightCycler 96 PCR platform (Roche). The primers used are listed in Additional file 1.

### Western blot analyses

Total cellular protein was extracted by lysis for 30 min on ice in RIPA buffer containing 150 mmol/l NaCl, 1% Triton X-100, 0.5% deoxycholate, 1% Nonidet P-40, 0.1% SDS, 1 mmol/l EDTA, 50 mmol/l TRIS (pH 7.6), protease inhibitor cocktail (10 μl/ml, Sigma Aldrich), and phosphatase inhibitor (10 μl/ml, Sigma Aldrich). Protein concentrations were determined by bicinchoninic acid protein assay (ThermoFisher Scientific, Darmstadt, Germany). Proteins were separated in SDS-PAGE gels and then wet-blotted to polyvinylidene difluoride (PVDF) membranes (Merck Millipore, Darmstadt, Germany). Membranes were blocked by 5% non-fat dry milk or BSA in TBS-T (150 mmol/l NaCl, 10 mmol/l TRIS, pH 7.6 and 0.1% TWEEN-20), washed several times, and then incubated with primary antibodies at 4 °C overnight. After several washings with TBS-T, membranes were incubated with horseradish peroxidase-conjugated secondary antibody at room temperature for 1 h. Membranes were then developed using Super Signal West Femto (ThermoFisher Scientific) or Western Bright Quantum (Biozym, Hessisch Oldendorf, Germany). α-tubulin was used as a loading control. Antibodies are listed in Additional file 1.

### Extraction and analysis of histones

Histones were acid-extracted according to a published protocol [21]. One microgram of each sample was used for Western blot analysis with 15% SDS-PAGE gels and PVDF membranes (Merck Millipore) as described above using antibodies listed in Additional file 1. Histone H3 was used as a histone loading control.

### Chromatin immunoprecipitation

ChIP-IT™ Express Kit (#53008, Active Motif, La Hulpe, Belgium) was used according to the manufacturer’s instructions. Rabbit Gamma Globulins (#31887, Invitrogen) served as a background control. Quantitative real-time PCR was used to determine enrichment of indicated gene regions at their transcriptional start site (TSS) as well as 2 kb upstream and downstream of each TSS. For a list of antibodies and primers, see Additional file 1.

### Generation of p57^KIP2^ knockout cells by gene editing

Cells were transfected by X-tremeGENE 9 DNA transfection reagent (Roche, Penzberg, Germany) with p57 Double Nickase Plasmid (sc-400444-NIC-2, Santa Cruz Biotechnology, Heidelberg, Germany) encoding a GFP marker, puromycin resistance, and two different sgRNAs targeting *CDKN1C* exon 1 and D10A mutant Cas9 (Nickase). Double Nickase control Plasmid (sc-437281) with non-targeting sgRNAs was used as a control. GFP expression allowed monitoring of transfection efficiency. Transfected cells were selected with 0.5 μg/ml puromycin for 5 days before single-cell seeding into 96-well plates. Genomic DNA was extracted from single-cell clones using QIAamp DNA Mini Kit (Qiagen). An amplicon spanning the sgRNA binding sites was amplified using HotStarTaq polymerase (Qiagen); PCR products were cloned into PCR4-TOPO TA Vector (450030, Invitrogen) and Sanger-sequenced. Mutant sequences were compared to the NCBI *CDKN1C* reference sequence (NG_008022.1). Successful knockout of p57^KIP2^ was verified by Western blot analysis.

## Results

### Knockdown of BRD4 exerts antineoplastic effects on UCCs

**Fig. 1:**
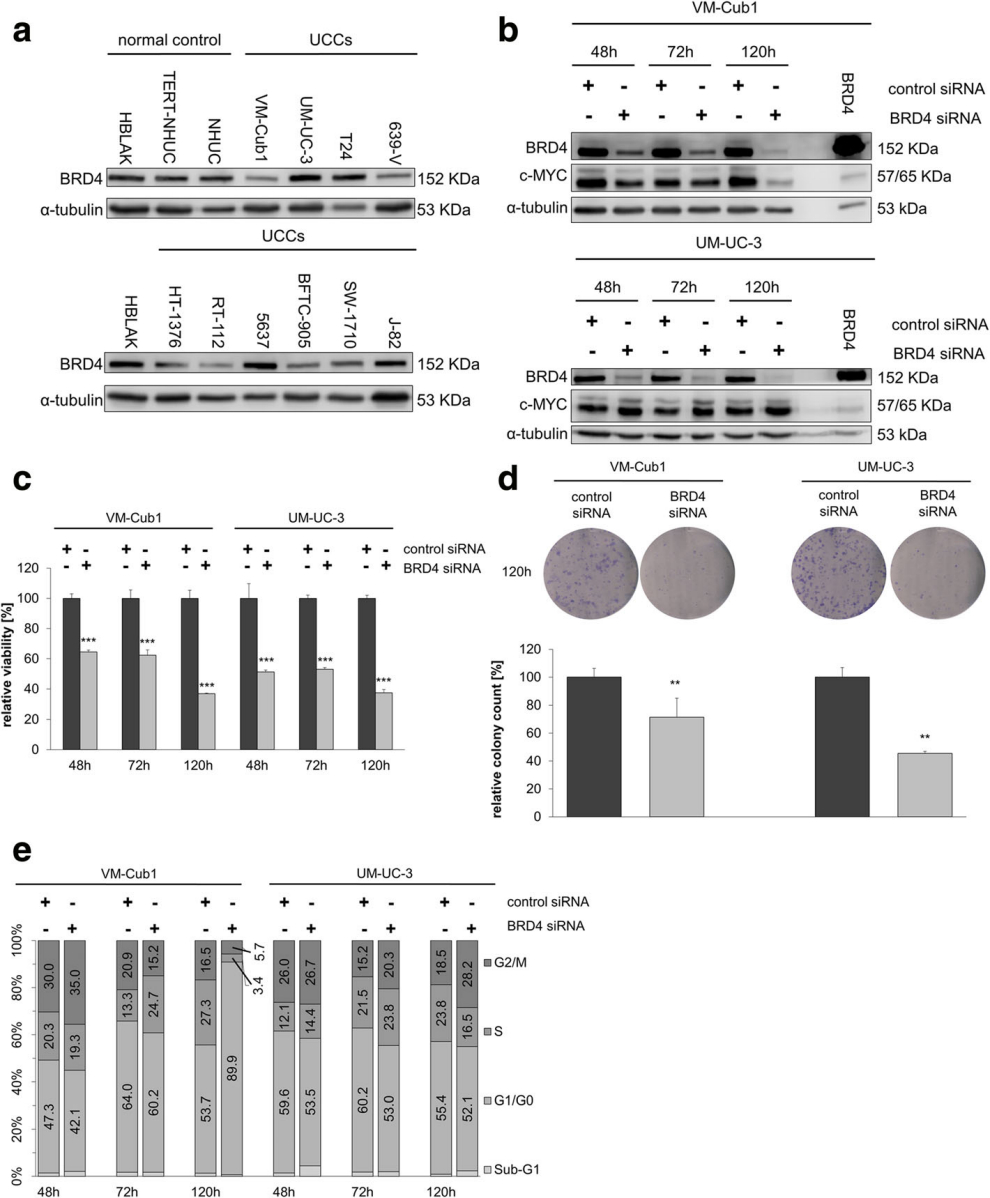
Effects of BRD4 knockdown on urothelial carcinoma cell lines. **a** Western blot analysis of BRD4 expression in 10 UCCs compared to the benign urothelial control cell lines HBLAK, NHUC, and NHUC-TERT. α-tubulin served as a loading control. **b** Western blot analysis of BRD4 and c-MYC expression after siRNA-mediated knockdown of BRD4 in VM-Cub1 and UM-UC-3 cells. Cells transfected with BRD4 expression plasmid served as a positive control, with 3 μg instead of 20 μg protein loaded. **c** Relative viability of VM-Cub1 and UM-UC-3 cells after BRD4 knockdown for 48, 72, and 120 h compared to treatment with control siRNA. Relative viability is displayed on the ordinate in percent of the control cells treated with non-targeting siRNA. Differences between control and targeting siRNA were analyzed using Student’s *t* test (***p ≤ 0.001). **d** Clonogenicity assays of VM-Cub1 and UM-UC-3 cells after BRD4 knockdown for 120 h and results of quantification (**p ≤ 0.01). **e** Flow cytometric cell cycle analysis of VM-Cub-1 and UM-UC-3 cells after BRD4 knockdown for 48, 72, or 120 h. Percentages of cells in the respective cell cycle phase are given

Since BRD4 is considered the most important target of the BET inhibitor JQ1 in various cancers, we evaluated BRD4 protein expression in a series of UCCs compared to the benign urothelial control cell lines HBLAK, TERT-NHUC, and NHUC (Fig. 1a). BRD4 was expressed in all tested UCCs at variable levels, in some UCCs more strongly than in the normal controls.

Next, we investigated by siRNA-mediated knockdown how dependent different UCCs are on BRD4 function and whether effects of siRNA knockdown differed from that by pharmacological inhibition in the same cell line. VM-Cub1 and UM-UC-3 cells were chosen for their very different phenotypes and have had been extensively characterized for their response to HDACi previously [3, 4]. As shown below, they were also differentially sensitive to JQ1 (Table 1 upper part). Efficient knockdown of BRD4 protein was confirmed at 48, 72, and 120 h post-transfection. Expression of c-MYC, a common target of BRD4, was strongly decreased in VM-Cub1 cells 120 h post-transfection, but not in UM-UC-3 (Fig. 1b).

Cell viability decreased following BRD4 knockdown in a time-dependent manner in both cell lines (Fig. 1c). Concordantly, clonogenic growth was significantly suppressed (Fig. 1d; *p* ≤ 0.01). Changes in cell cycle distribution intensified over time; VM-Cub1 cells became arrested in G0/G1, whereas UM-UC-3 cells accumulated in G2/M (Fig. 1e). Only minor increases in subG1 fractions were observed.

### UCCs differ in sensitivity to pharmacological BET inhibition

Next, we investigated the dose response of eight different UCCs, including VM-Cub1 and UM-UC-3, to JQ1. JQ1 diminished viability in all cell lines, but they clearly fell into two groups, with low and high sensitivity. Calculated IC_50_ values were below 0.5 μM in the first group, but well above 1 μM in the second group (Table 1). Notably, at the highest concentration of JQ1 (25 μM) tested, a sizeable fraction of cells survived the treatment. This fraction correlated only partially with the respective IC_50_, indicating that JQ1 alone acted rather in a cytostatic fashion. No correlation was seen between sensitivity to JQ1 and BRD4 protein expression or any other previously investigated characteristics of the cell lines. Benign control cells generally responded to low concentrations of JQ1 (IC_50_ 0.26–0.4 μM).

### BET inhibitor JQ1 and class I HDAC inhibitor Romidepsin synergistically inhibit UCC proliferation and clonogenic growth

**Fig. 2:**
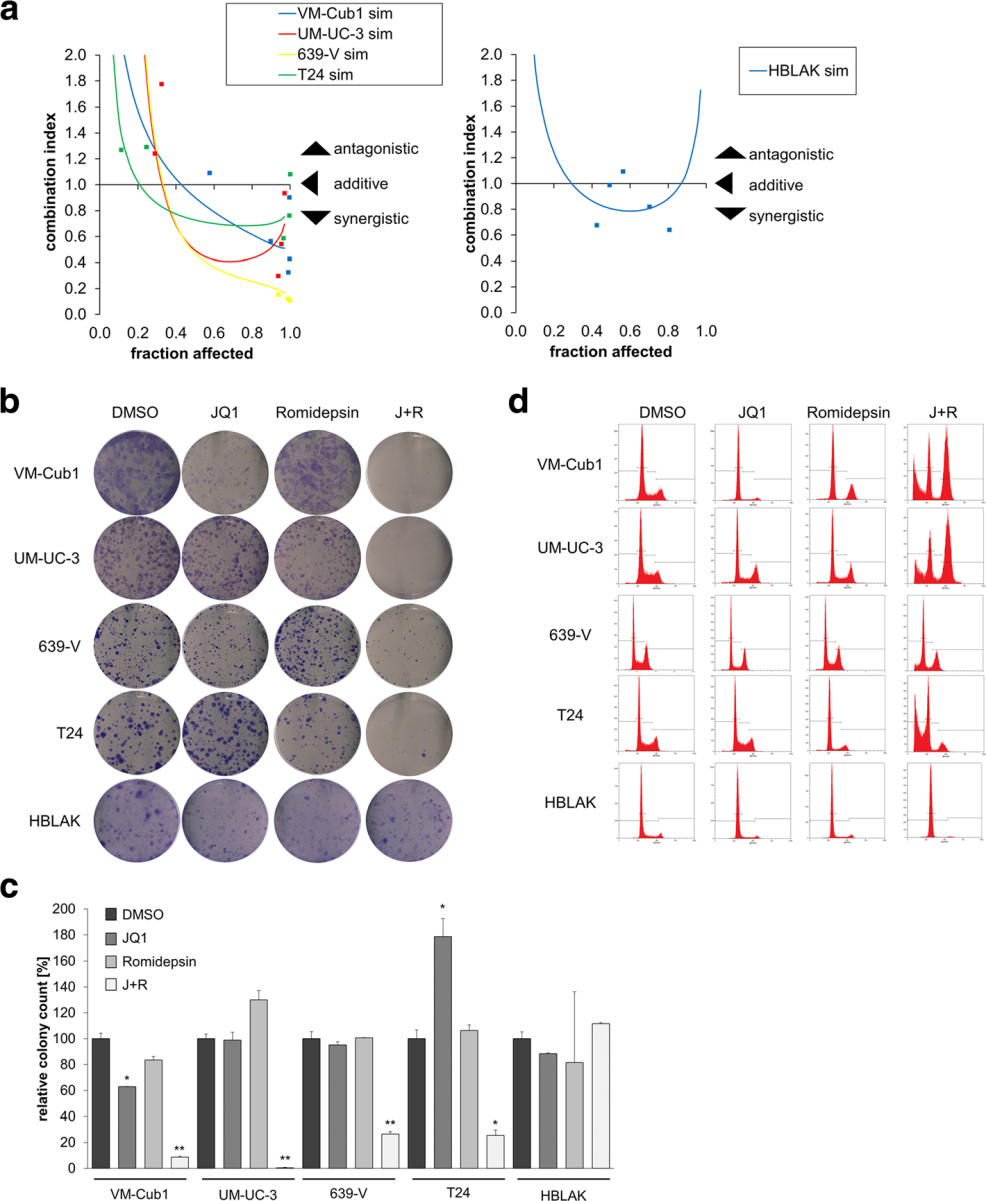
Effects of combined treatment with JQ1 and Romidepsin on proliferation and clonogenic growth of UCCs. **a** Combination index (CI/Fa) plots for the combination of JQ1 and Romidepsin. Cell viability was measured at five constant dose ratio experimental data points by ATP assay after 72-h treatment. CI plots were then generated using CompuSyn software. CI < 1 indicates synergism. Benign HBLAK cells were compared to four UCCs. **b** Clonogenicity assay following treatment for 48 h with JQ1, Romidepsin, or both compounds compared to DMSO as indicated in Table 1 (lower part). **c** Quantification of colony counts from clonogenicity assays. Differences between control and treated cells were analyzed using Student’s *t* test (**p ≤ 0.01, *p ≤ 0.05). **d** Flow cytometric cell cycle analyses following the indicated treatment for 48 h in four different UCCs and in HBLAK cells. See Additional file 3 for cell numbers in the respective cell cycle phases

Cell viability results from dose-response curves for JQ1, and Romidepsin were used to generate combination index plots (Fa/CI-Plots; fraction affected/combination index; Fig. 2a) by the Chou-Talalay method [19]. Strong synergisms (CI < 1) were detected for all four UCCs at effect rates (Fa; percentage of dead cells) from 0.2 (T24) to 0.4 (UM-UC-3). Synergies intensified with increasing effect rates. Intriguingly, the strongest synergies were seen in the less JQ1-sensitive 639-V and UM-UC-3 cells. Similarly, primary cancer cell cultures established from patient tissues responded strongly towards combined treatment resulting in strong synergies (Additional file 2). In contrast, only a limited synergy of the combined treatment was seen with benign HBLAK cells (Fig. 2a).

Next, we compared the effects of single and combined treatment on cell proliferation and apoptosis. Dosages for the combined treatment were chosen from synergistic combinations and mostly below individual agent IC_50_s (Table 1). Single treatment with JQ1 alone diminished clonogenic growth of the more sensitive VM-Cub1 cells (Fig. 2b), whereas the less sensitive UM-UC-3 cells readily formed colonies. Clone formation by benign HBLAK cells was rather unaffected. Next, we evaluated the combination of JQ1 and Romidepsin in two more (VM-Cub1 and T24) and two less (UM-UC-3 and 639-V) JQ1-sensitive UCCs and in HBLAK as a benign urothelial control. The combination treatment synergistically suppressed long-term proliferation of all four UCCs (at least 4-fold compared to DMSO; Fig. 2b, c), much more strongly than each inhibitor alone at the same doses (at least 3.5-fold compared to single treatments; Fig. 2b, c). Remarkably, no significant inhibition of clonogenic growth was observed in HBLAK cells.

To characterize the cellular effects of the drug combination in more detail, we performed cell cycle analysis by flow cytometry. Treatment with JQ1 alone led to accumulation of cells in the G0/G1-phase and a decreased S-phase fraction in most UCCs (Fig. 2d, Additional file 3), except in UM-UC-3, where an increased G2/M fraction was observed. The subG1 fraction increased slightly in several, albeit not in all UCCs. HBLAK cells were likewise arrested in G0/G1, with decreased S-phase fraction, but no increased subG1 fraction. Cell cycle distribution changes after treatment with JQ1 were very similar to those after BRD4 knockdown (see Fig. 1e). Likewise, UCCs responded towards single treatment with Romidepsin by accumulating in G0/G1 or G2/M (Fig. 2d) in accord with our previous results [4].

**Fig. 3:**
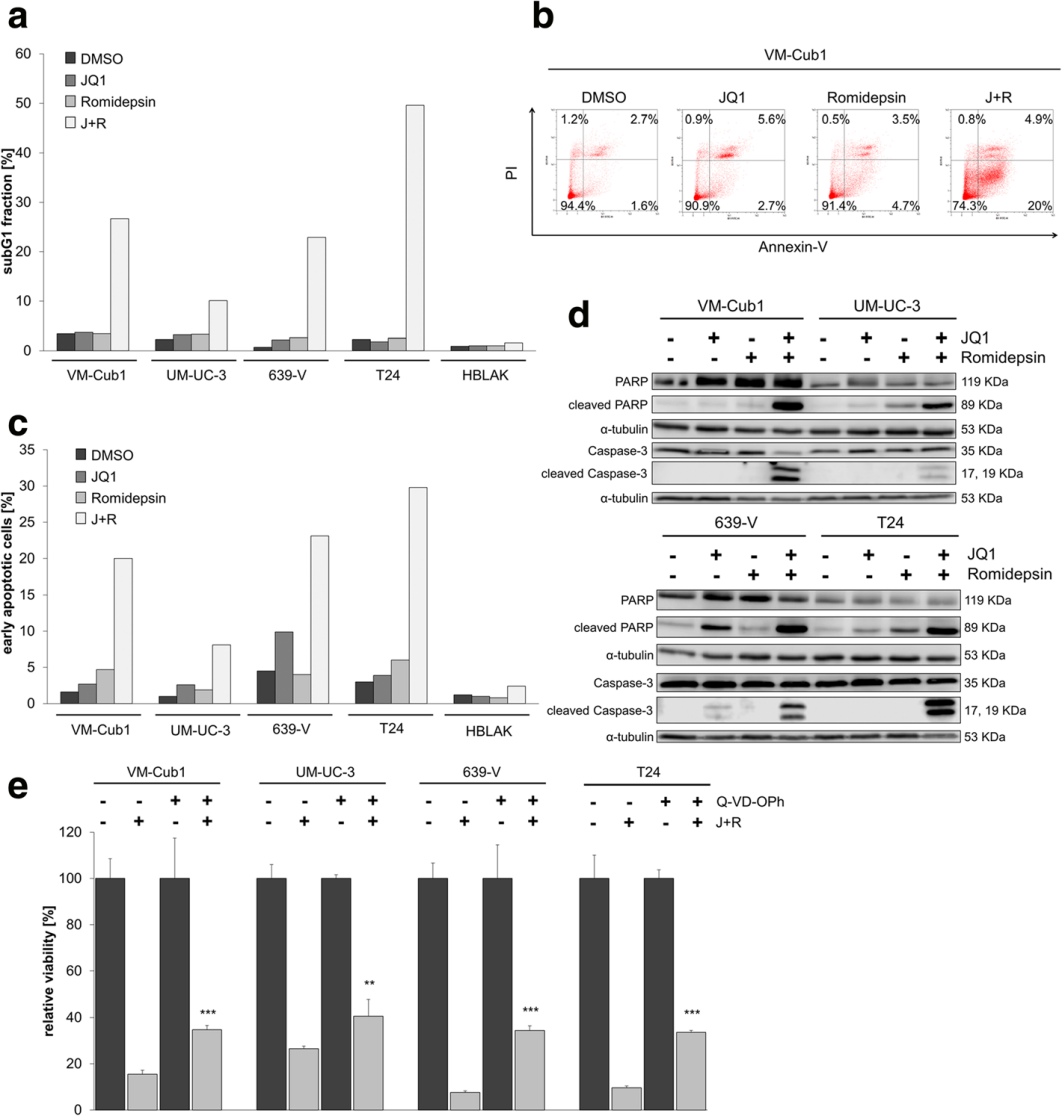
Induction of apoptotic cell death by combined treatment with Romidepsin and JQ1. **a** Increase of subG1 fraction by combination treatment as determined by flow cytometry. **b** Flow cytometric analysis of UCCs with indicated treatment (Table 1 lower part) after combined staining with PI and Annexin V. Percentages of viable (lower left), early (lower right), or late (upper right) apoptotic and necrotic (upper left) VM-Cub1 cells subsequent to indicated treatments. **c** Percentage of early apoptotic cells as measured by Annexin V staining for all UCCs and HBLAK control cells are displayed in bar graphs for the respective treatment. **d** PARP and Caspase-3 cleavage 48 h after treatment assessed by Western blot analysis. **e** Four UCCs received the combination treatment with or without the Pan-Caspase inhibitor Q-VD-Oph at 30 μmol/l. All cells received the same concentration of DMSO. Cell viability displayed on the ordinate was measured by ATP assay after 48 h. Differences between J + R treatment and J + R treated plus Caspase inhibitor were analyzed using Student’s *t* test (***p ≤ 0.001, **p ≤ 0.01)

In contrast, combination treatment resulted consistently in a strong increase of the subG1 fraction across all four UCCs (Fig. 3a), whereas the number of S-phase cells was reduced dramatically, indicating cell cycle inhibition and cell death (Fig. 2d, Additional file 3). Although the cell lines reacted rather uniformly in so far, they differed in the cell cycle phase, in which they became arrested. Whereas VM-Cub1 and UM-UC-3 cells showed a strong G2/M increase with decreased G0/G1 cells, 639-V and T24 cells displayed an increased G0/G1 fraction (Fig. 2d, Additional file 3). HBLAK cells arrested in G0/G1 with a remarkable decrease of the S-phase and G2/M fractions. Notably, the combination induced a subG1 fraction much more prominently than either agent on its own in UCC, but not in HBLAK cells (Fig. 3a).

### Combination treatment causes apoptotic cell death

The increased subG1 fractions and according morphological changes in cell morphology indicated that combination treatment elicited pronounced apoptosis. Concordantly, the number of early apoptotic cells determined by Annexin V staining (Fig. 3b, c and Additional file 4) as well as cleaved Caspase 3 and cleaved PARP were significantly enhanced (Fig. 3d). These markers of apoptosis were not or only weakly induced by either a single drug treatment. Of note, induction of apoptosis was less pronounced in UM-UC-3 compared to the other UCCs. Moreover, cytotoxicity of the combination treatment was partly prevented by the Pan-Caspase inhibitor Q-VD-Oph in all four UCCs (Fig. 3e) indicating that the combination treatment efficiently elicited caspase-dependent apoptosis.

**Fig. 4:**
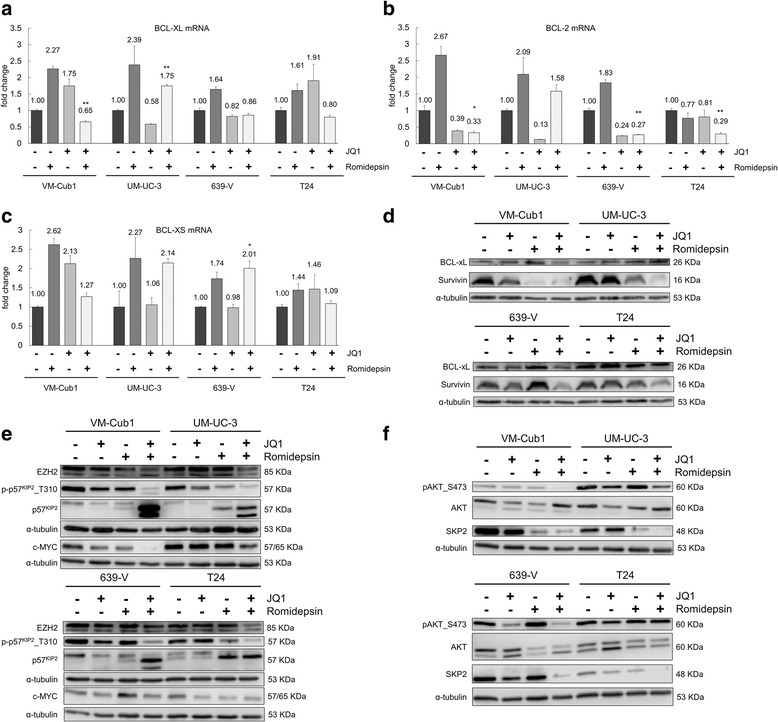
Changes in expression of apoptotic regulators and cellular signaling after combined treatment with Romidepsin and JQ1. Relative mRNA expression levels of antiapoptotic *BCL-XL* (**a**) and *BCL-2* (**b**) and proapoptotic *BCL-XS* (**c**). mRNA levels were measured by qRT-PCR and normalized to the expression of *TBP*. Fold change compared to DMSO control 48 h after treatment is displayed on the ordinate. Differences between combination treatment samples and untreated controls were analyzed using Student’s *t* test (**p ≤ 0.01, *p ≤ 0.05). **d** Protein expression of antiapoptotic BCL-XL and Survivin after combination treatment for 48 h assessed by Western blot. **e** Protein expression of c-MYC, EZH2, p57^KIP2^, and phospho-p57^KIP2^ (Thr310) assessed by Western blot analysis. **f** Phosphorylated AKT (S473) and expression of AKT and SKP2 was detected by Western blot analysis

Concurringly, mRNA expression of the antiapoptotic regulators BCL-XL (Fig. 4a) and BCL-2 (Fig. 4b) was diminished by combination treatment in UCCs, again with the exception of UM-UC-3. Expression of BCL-XS was either increased or remained unchanged (Fig. 4c). Similarly, the antiapoptotic BCL-XL and Survivin proteins were generally downregulated by the combination treatment (Fig. 4d). These anti-apoptotic proteins were not consistently downregulated by either single agent and in some cases actually induced. Thus, in contrast to single drug treatments, combined treatment led to stronger and more consistent downregulation of antiapoptotic regulators. This downregulation was less pronounced in UM-UC-3, presumably accounting for the weaker apoptotic response in this cell line.

Expression of c-MYC and EZH2, which have previously been identified as targets of BRD4 knockdown and JQ1 treatment mediating cell cycle arrest and apoptosis [10], were likewise clearly diminished following combined treatment (Fig. 4e).

### JQ1 and Romidepsin synergistically induce p57^KIP2^ by various mechanisms

Since the cell cycle regulator protein p57^KIP2^ (encoded by the *CDKN1C* gene, hereafter p57) had been reported to mediate synergistic induction of apoptosis by combined HDAC and BET inhibition in pancreatic ductal adenocarcinoma [8], we evaluated its expression in four UCCs after treatment. p57 mRNA was induced between 17-fold (T24) and up to 1000-fold (VM-Cub1) by combination treatment. It was also induced regularly, but to a smaller extent, by Romidepsin, but not consistently by JQ1 (Additional file 5). Western blot analysis revealed a double band around the predicted molecular weight, of which especially the lower band was strongly induced by the combination treatment (Fig. 4e). The upper band likely reflects a phosphorylated isoform generated by active AKT protein kinase which is destined to degradation via a SKP2-containing E3 ubiquitin ligase complex. Accordingly, a (T310) phospho-specific p57 antibody demonstrated loss of phosphorylated p57 following combination treatment, indicating accumulation of the more stable unphosphorylated p57 protein (Fig. 4e).

Accordingly, active pAKT was diminished in three of four cell lines by JQ1 single treatment, but particularly strongly by combined treatment (Fig. 4f). SKP2 expression, too, was strongly downregulated in all four cell lines by the combination treatment. VM-Cub1 cells displayed the strongest induction of p57 protein and mRNA and the most pronounced decrease in its negative regulators c-MYC, pAKT, and SKP2. Conversely, T24 cells displayed the weakest increase of p57 protein and pAKT levels remaining unchanged. These findings suggest that, in addition to increased transcription of *CDKN1C*, stabilization of p57 contributes to its accumulation. Of note, reduced activity of AKT by combined treatment is likely to diminish pro-survival signaling in UCCs by other pathways as well.

STAT3 activation may also be involved in regulation of cell cycle progression and anti-apoptotic response and had been reported to be inactivated by combined HDAC and BET inhibition in pancreatic ductal adenocarcinoma [8]. However, we neither observed significant changes in STAT3 phosphorylation nor expression after combined treatment with JQ1 and Romidepsin (Additional file 6).

### Gene editing of *p57*^*KIP2*^ in UCC

**Fig. 5:**
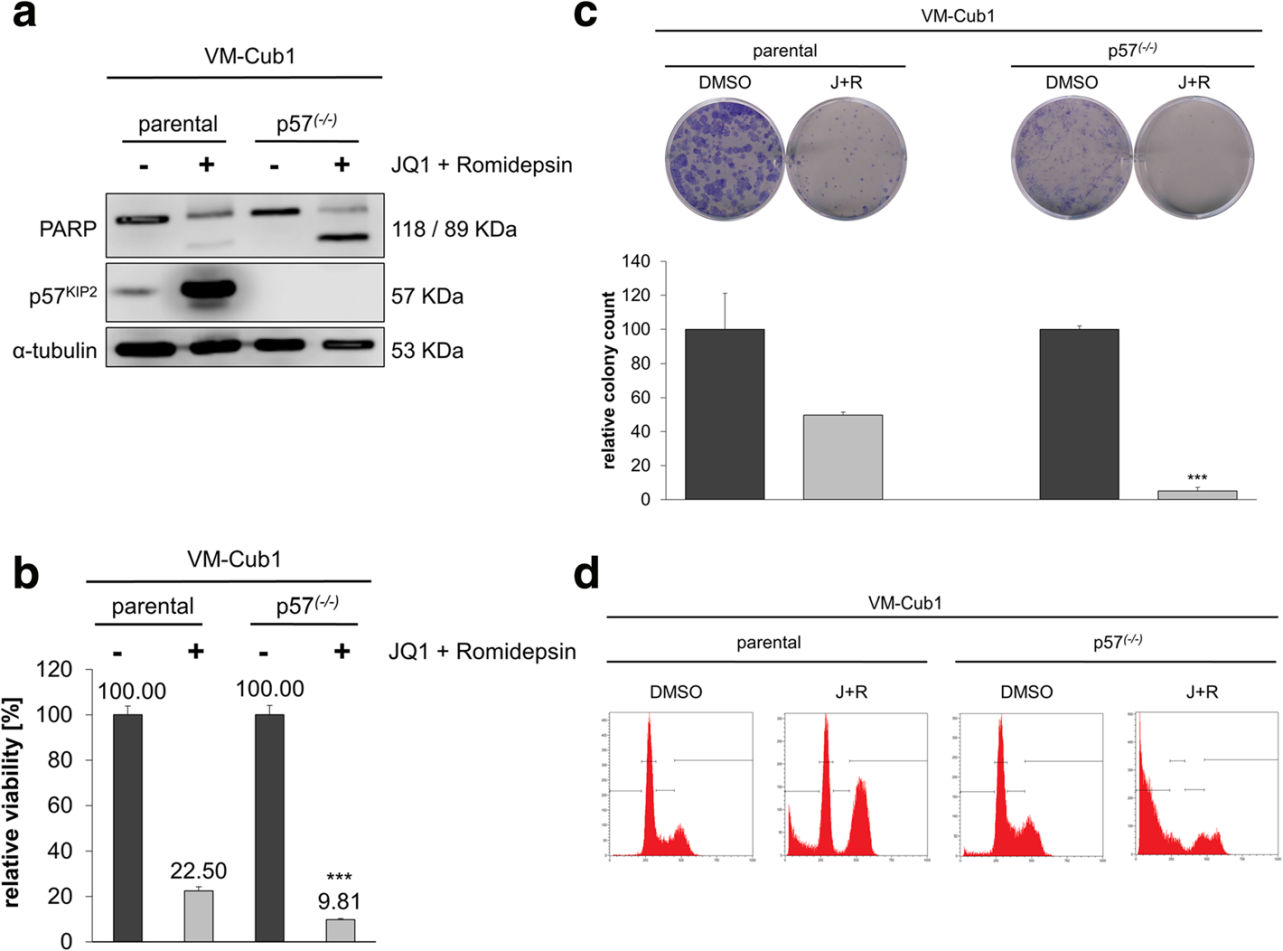
Effect of *p57*^*KIP2*^ gene knockout on induction of apoptosis by combination treatment. **a** Protein expression of p57 and PARP1 in parental and knockout VM-Cub1 cells with or without combination treatment. **b** Relative viability of VM-Cub1 parental and *p57* knockout cells after combination treatment. Differences between samples with combination treatment of parental and knockout cells were analyzed using Student’s *t* test (***p ≤ 0.001). **c** Examples of clonogenicity assay, with quantified colony counts (***p ≤ 0.001) and **d** cell cycle analysis for the indicated cells following combined inhibitor treatment. See Additional file 3 for cell numbers in the respective cell cycle phases

To investigate to which extent p57 contributes to apoptosis induction by combined treatment in UCCs, we generated VM-Cub1 *p57* knockout clones by CRISPR/Cas9-mediated gene editing. In contrast to the parental cells, p57 protein remained undetectable in cell clones with successful knockout even after combined treatment (Fig. 5a). Surprisingly, however, *p57* knockout did not rescue the cells from induction of cell death; viability was instead further decreased (Fig. 5b) and cleaved PARP was enhanced (Fig. 5a). Colony formation assays displayed concurring results (Fig. 5c), as did cell cycle analysis. Double treatment of *p57* knockout cells disrupted the cell cycle completely, and the majority of cells died rapidly (Fig. 5d, Additional file 3).

### Combination treatment strongly increases histone acetylation around transcriptional start sites

**Fig. 6:**
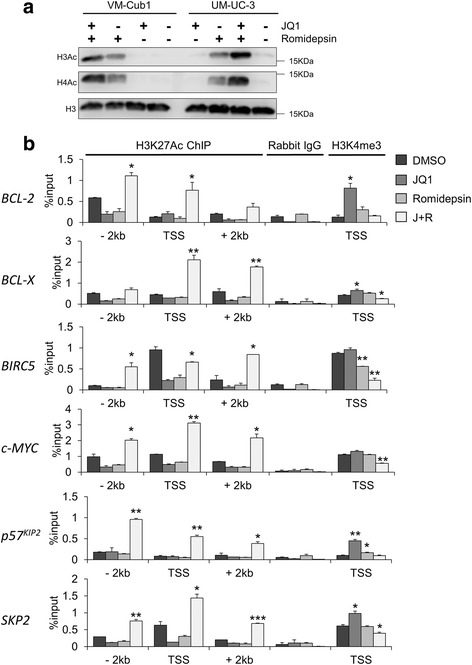
Changes in histone modifications elicited by combination treatment. **a** Changes in H3 and H4 acetylation were detected after single and combined treatment in VM-Cub1 and UM-UC-3 cells by acid histone extraction and Western blot analysis. Total histone 3 served as a loading control. **b** Chromatin immunoprecipitation analysis of H3K27 acetylation at transcriptional start sites (TSS) as well as 2 kb upstream (− 2 kb) and 2 kb downstream (+2 kb) of *BCL-2*, *BCL2L1/BCL-X*, *BIRC5/Survivin*, *c-MYC*, *CDKN1C/p57* and *SKP2 genes* after single and combined treatment of VM-Cub1 cells. Immunoprecipitated DNA amounts measured by qPCR for each condition are expressed as percentage of input DNA. Additionally, H3K4 trimethylation was analyzed at the TSS of the same genes. Rabbit IgG served as a background control. Differences between samples with combination treatment and DMSO treated cells were analyzed using Student’s *t* test (***p ≤ 0.001, **p ≤ 0.01, *p ≤ 0.05)

To follow the effects of the drug combination on the chromatin level, we analyzed changes in histone acetylation. Overall, acetylation of histones H3 and H4 increased after treatment with Romidepsin alone, but was further enhanced by combination treatment (Fig. 6a). We further analyzed H3K27 acetylation and H3K4 trimethylation, which are established markers for active transcription, around the transcriptional start sites of six genes affected by the drug combination using chromatin immunoprecipitation (ChIP). ChIP results for acetylated H3K27 revealed a broad and strongly enhanced enrichment of acetylation after combined treatment encompassing at least 2 kb upstream of the transcriptional start site (TSS) to 2 kb downstream of the TSS of all six investigated genes (Fig. 6b). By comparison, treatment with Romidepsin alone did not significantly increase H3K27 acetylation compared to DMSO controls. Strikingly, H3K4 trimethylation was increased at the TSS of BCL2, *CDKN1C*/p57, and SKP2 genes after treatment with JQ1 alone, but decreased, mostly significantly, at the TSS of genes downregulated by the combination treatment.

## Discussion

In this study, we examined the efficacy of a combination treatment with small-molecule inhibitors of chromatin regulators in urothelial carcinoma cell lines. In previous studies, we had identified HDAC class I-specific inhibitors like Romidepsin to be the most efficient HDAC inhibitors in UC. However, as this treatment did not induce apoptosis straightforwardly and did not appear particularly selective for tumor cells, we searched for a second drug for an improved combination therapy [2, 4]. Here, we combined Romidepsin with the BET inhibitor JQ1, which had been proposed to synergize with HDACi in several cancer types and to induce a canonical apoptotic response [22]. To our knowledge, the particular combination of Romidepsin and JQ1 has only been investigated by Jostes et al. in testicular cancer cell lines. These authors reported about reduced tumor burden even at lower and less frequent doses in xenograft experiments [6]. Other studies to date applied mainly pan-HDAC inhibitors like panobinostat or SAHA [23–25].

The results of our current study clearly demonstrate a significant synergistic effect on cell proliferation and prominent apoptotic cell death for the combination of the BET protein inhibitor JQ1 and the HDAC class I-specific inhibitor Romidepsin. As UC cells are quite resistant to apoptosis induction under many conditions, we were especially interested in characterizing these cellular effects.

A pioneer study on the efficacy of single treatment with JQ1 alone in two related UC cell lines (T24 and EJ) provided first direct evidence that UC cells may be dependent on BRD4 [10]. shRNA mediated knockdown in the respective cells, decreased cell viability in a time-dependent manner (72 h), and induced cell cycle arrest in G0/G1 phase. In addition, in vivo tumor growth was reduced by BRD4 inhibition in a xenograft mouse model. We also found BRD4 overexpressed in some of the 12 investigated UC cell lines compared to benign urothelial cells, but overexpression was not uniformly observed, indicating that T24 may not be representative for all UC cell lines. Indeed, in our study, siRNA-mediated knockdown of BRD4 in both VM-Cub1 and UM-UC-3 cells resulted in a time-dependent reduction of cell viability and long-term proliferation, but the cells responded differentially with respect to cell cycle arrest. VM-Cub1 cells displayed the expected arrest in G0/G1, whereas UM-UC-3 arrested in G2/M. In many further functional assays, we likewise observed that UM-UC-3 cells responded differently or less efficiently, e.g., with regard to induction of apoptosis. Similar heterogeneity of BRD4 expression and response to BRD4 inhibition may occur in cancer patients due to the pronounced heterogeneity of urothelial carcinoma. This prompted us to characterize the response of UC towards single and combined treatment more comprehensively across different cell lines.

Indeed, our investigations on the cellular effects of pharmacological BRD4 inhibition in eight UC cell lines chosen to represent the heterogeneity of urothelial carcinoma demonstrated that the previously reported results on T24 [10] cannot be simply extended to all UC cell lines. Instead, dose-response curves for JQ1 in eight UC cell lines demonstrated that they could be clearly classified into two groups with low and high sensitivity and that not all UCCs are as sensitive as T24. The differential sensitivity was not related to BRD4 protein expression or any other obvious characteristics of the cell lines and remains to be further investigated.

Like T24 in the study by Wu et al. [10], most cell lines accumulated in G0/G1 following treatment with IC_50_ doses of JQ1 (0.18–6.8 μM), but UM-UC-3 accumulated in G2/M. At these doses, we detected only a slight increase of the subG1-fraction. Comparable results were reported for colon cancer and medulloblastoma cells, which arrested in G0/G1 after treatment with 0.1–0.5 μM JQ1 [14, 26]. However, in contrast to various other cancer cell types, JQ1 treatment alone neither significantly inhibited long-term proliferation of UCCs nor induced significant apoptosis suggesting that UC cells are generally less sensitive towards JQ1 than other tumor types [8, 14, 24, 27] and recover over time. Benign urothelial cells also arrested at rather low concentrations, but no apoptosis was observed and long-term proliferation ability was retained.

Using the Chou-Talalay method [19], we determined doses for the combined treatment with JQ1 and Romidepsin with synergistic effects on cell viability at doses mostly lower than the IC_50_ concentrations of either single treatment. Importantly, all UC cell lines, including those with low sensitivity to JQ1 alone like UM-UC-3 and 639-V, as well as primary tumor cultures, responded strongly to the combination treatment. The combination of JQ1 with Romidepsin was clearly more efficacious in inhibiting proliferation of UCCs than JQ1 alone. Moreover, the combination elicited apoptosis much more efficiently than either JQ1 or Romidepsin. Benign control cells were strikingly less affected suggesting that the combined treatment might achieve a higher tumor specificity with lower toxic side effects for normal cells than single treatment with Romidepsin [4]. Accordingly, long-term proliferation was not affected in HBLAK cells, but was very strongly diminished in all cancer cell lines, again irrespective of their sensitivity towards JQ1 alone. The cell cycle was heavily disturbed, and we observed very strongly increased subG1 fractions as well as high numbers of early apoptotic cells subsequent to combination treatment in UCCs, but not in benign HBLAK cells. Similar synergistic effects on proliferation and apoptosis have been reported in pancreatic cancer, neuroblastoma, and AML cells by combination treatment using JQ1 with the pan-HDAC inhibitors SAHA (vorinostat) or panobinostat [8, 23, 24] and in melanoma cells by panobinostat and BET inhibitor I-BET151 [7]. Borbeley et al. also combined the class I-specific inhibitor mocetinostat with JQ1 for treatment of breast cancer cells [27]. Interestingly, normal melanocytes, embryonic fibroblasts, and normal hematopoietic progenitor cells were spared from apoptosis similar to HBLAK cells, again emphasizing the increased tumor specificity of the combination [7, 23, 24].

Molecular biomarkers and application of the caspase-inhibitor Q-VD-OPh confirmed that the combination treatment, other than treatment with either inhibitor alone, efficiently induced canonical caspase-dependent apoptosis in UC cells. Similar changes in apoptosis-related proteins were found in pancreatic cancer [8], neuroblastoma [23], melanoma [7], and AML cells [24]. Anti-apoptotic proteins like BCL-2 may be induced by activated STAT3 [25, 28], and in pancreatic adenocarcinoma cells, the combination of JQ1 and SAHA diminished STAT3 phosphorylation to downregulate BCL-2 [8]. However, in UC cells, we observed neither diminished phosphorylation nor decreased expression of STAT3, again indicating that the mechanisms mediating cellular effects may differ between cancer entities. Accordingly, gene expression profiles following treatment with BETi and HDACi drug combination in studies on different cancer cell types each describe hundreds of differentially expressed genes, with limited overlap, but converging on a cellular response resulting in cell cycle arrest and apoptosis [23, 27]. These relatively uniform ultimate cellular effects thus appear to be driven by cell type-dependent transcriptional programs.

In addition, suppression of AKT-mediated survival signaling is likely to contribute to the observed cell death. Diminished AKT kinase activity may especially contribute to the dramatic overexpression of p57^KIP2^ via stabilization of the protein following decreased phosphorylation priming for SKP2-mediated degradation [29, 30], as especially non-phosphorylated p57^KIP2^ accumulated. SKP2 itself was as well strongly diminished by the treatment, presumably further contributing to p57 accumulation. In accordance with other studies reporting p57^KIP2^ as a prominent factor induced in treated cells [6, 8], we found both its mRNA and protein strongly elevated.

The function of p57 with regard to regulation of apoptosis is controversial. Obviously, p57 can prevent or promote apoptosis dependent on the cellular context [31]. Promotion of apoptosis may occur by p57 translocation into mitochondria to trigger the intrinsic apoptotic pathway [32]. In pancreatic cancer cells, depletion of *p57* by shRNA decreased apoptotic markers induced by JQ1 and SAHA combination treatment; Cas9-mediated knockout of *Cdkn1c* in mice significantly diminished apoptosis of combination-treated animals [8]. In an analogous approach, we generated *p57* knockout UC cells by gene editing. Intriguingly, this knockout did not rescue the cells from apoptosis induced by combination treatment. This finding underlines the context-dependent function of p57 on the one hand. On the other hand, it supports again the idea that compounds like JQ1 targeting general transcriptional regulators do rarely act through their effect on a single gene but elicit broad effects on the transcriptome which are cell type-dependent due to the respective transcriptional programs [5]. For instance, in other studies, suppression of c-MYC was proposed to be the central event mediating effects in the treated cells, but forced overexpression of this supposedly crucial target gene failed to rescue all phenotypes [5]. In this respect, we note that downregulation of c-MYC was observed in all UCCs treated with the drug combination, but that its extent varied.

Recent publications have suggested a mechanism for the downregulation of oncogenic and anti-apoptotic factors like c-MYC or BCL-2 after combined treatment with HDAC and BET inhibitors [23, 33]. HDAC inhibitor treatment induces histone hyperacetylation, as also observed in UC cells. Genes undergoing hyperacetylation of H3K27 within a 5 kb region around their transcriptional start site by this treatment appear to become particularly dependent on binding of BET proteins like BRD4 to their regulatory regions, including enhancers, to promote gene transcription. Accordingly, genes activated by HDAC inhibitor treatment were reported to be enriched for BRD4 [33]. Abolishment of BRD4 binding by JQ1 then leads to their transcriptional inactivation. This mechanism has been proposed for combined treatment with 4SC-202, Entinostat, or mocetinostat [27, 33], all of which predominantly target class I HDAC isoenzymes, similar to Romidepsin. Indeed, Romidepsin alone resulted also in activation of anti-apoptotic factors like BCL-2 and BCL-X in UC cells, suggesting that this mechanism also applies in our experimental setting. Accordingly, ChIP analysis revealed broad H3K27 hyperacetylation around the transcriptional start sites of all investigated genes after combined treatment, which would render them sensitive to transcriptional repression by JQ1. Consequently, H3K4 trimethylation, a marker for transcriptionally active genes, was significantly reduced at the TSS of four genes downregulated by combination treatment in UC cells. Notably, Mishra et al. [33] reported globally increased H3K4me3 at the TSS of genes upon treatment with the HDAC inhibitor 4SC-202, which—different from Romidepsin—additionally inhibits the LSD1 histone demethylase.

## Conclusions

In conclusion, we have demonstrated that the combination of JQ1 and Romidepsin exerts strong synergistic antineoplastic effects on urothelial carcinoma cells. Due to the strong synergy, the applied doses were well below the IC_50_ doses of the single treatments and affected benign control cells only mildly. UC cells are often particularly resistant towards induction of caspase-dependent apoptosis [34]. Therefore, this novel combination treatment constitutes a promising approach for a small-molecule therapy of UC with reduced toxic side effects to normal cells.

## Additional files

Additional file 1: Information on primer sequences and antibodies. Sequence information and amplicon sizes for qRT-PCR and ChIP qPCR primers as well as product information and dilution of applied antibodies are given. (PDF 271 kb)

Additional file 2: Data on synergistic effects on cell viability of primary cancer cells by combined treatment with Romidepsin and JQ1. (a), (b) Relative viability of primary cultures 1 and 2 established from primary tumor tissue of two different patients after single or combined treatment for 48 h. Relative viability is displayed on the ordinate in percent of the control cells treated with DMSO. (c) Primary culture 2 was used for an extended dose response curve analysis after 24 h to perform Chou-Talalay calculations (***p ≤ 0.001, **p ≤ 0.01, *p ≤ 0.05). (d) Combination index (CI/Fa) plot for the combination of JQ1 and Romidepsin. Cell viability was measured at five constant dose ratio experimental data points by ATP assay for primary culture 2. CI plots were then generated using CompuSyn software. CI < 1 indicates synergism. (PDF 293 kb)

Additional file 3: Detailed flow cytometry results from cell cycle analysis. Displayed are percentages of cells in the indicated cell cycle phase as measured by flow cytometric cell cycle analyses following the indicated treatment for 48 h in four different UCCs and in HBLAK cells (Fig. 2d) or in VM-Cub1 knockout cells (Fig. 5d). (PDF 177 kb)

Additional file 4: Data on induction of apoptotic cell death by combined treatment with Romidepsin and JQ1. Induction of apoptotic cell death by combined treatment with Romidepsin and JQ1. Flow cytometric analysis of UCCs with indicated treatment after combined staining with PI and Annexin V. Percentages of viable (lower left), early (lower right), or late (upper right) apoptotic and necrotic (upper left) VM-Cub1 cells subsequent to indicated treatments. (PDF 1052 kb)

Additional file 5: Data on expression changes of of *p57*^*KIP2*^ mRNA by combination treatment in UC cells. Relative mRNA expression levels of *p57*^*KIP2*^ mRNA after single and combined treatment with Romidepsin and JQ1. mRNA levels were measured by qRT-PCR and normalized to the expression of TBP. Fold change compared to DMSO control 48 h after treatment is displayed on the ordinate. (PDF 100 kb)

Additional file 6: Data on STAT3 activation and expression after combination treatment in UC cells. Phosphorylated and total STAT3 protein was detected by Western blot analysis in four UC cell lines cells after indicated treatment. α-tubulin served as an additional loading control. (PDF 216 kb)

## Abbreviations

BET: Bromodomain and extra-terminal
CI: Combination index
Fa: Fraction affected
HDAC: Histone deacetylase
HDACi: HDAC inhibitor
NHUC: Primary normal human urothelial cells
PVDF: Polyvinylidene difluoride
TSS: Transcriptional start site
UC: Urothelial carcinoma
UCC: Urothelial carcinoma cell
