# Supplementary material for: Combined inhibition of BET proteins and class I HDACs synergistically induces apoptosis in urothelial carcinoma cell lines
Source: Clin Epigenetics. 2018 Jan 4;10:1. doi: 10.1186/s13148-017-0434-3 (PMC5755363; doi:10.1186/s13148-017-0434-3)
Supplement: Supplementary file 1 — Information on primer sequences and antibodies. Sequence information and amplicon sizes for qRT-PCR and ChIP qPCR primers as well as product information and dilution of applied antibodies are given. (PDF 271 kb) [file 13148_2017_434_MOESM1_ESM.pdf]

Additional file 1

qRT-PCR Primers			
Target	5‘-3‘ sequence forward	5‘-3‘ sequence reverse	Product size [bp]
p57 / *CDKN1C*	GCGGCGATCAAGAAGCTG	CGACGACTTCTCAGGCGC	71
BCL-Xl	TAAACTGGGGTCGCATTGTG	AGGTAAGTGGCCATCCAAGC	118
BCL-Xs	GCAGTAAAGCAAGCGCTGAG	GTTCCACAAAAGTATCCTGTTCAAAG	139
BCL-2	CCTGTGGATGACTGAGTACCTG	CAGAGGCCGCATGCTGGG	106
TBP	ACAACAGCCTGCCACCTTA	GAATAGGCTGTGGGTCAGT	119

ChIP qPCR Primers			
Target	5'-3' sequence forward	5'-3' sequence reverse	Product size [bp]
BCL-2 TSS	CCGTTGCTTTTCCTCTGGGA	CATCTCCCGCATCCCACTC	126
BCL-2 TSS +2kb	TGTGGTGTGCTTCTTGACATCT	GTGCTTCCTTTTATAGCCTCTTGG	118
BCL-2 TSS -2kb	TGTGGGAGTGTGTGTGTCGC	GAGCGCCGGTAACACAACGT	93
BCL-X TSS	GACTCAGTGAGTGAGCAGGTGT	CTCCAGCTGTATCCTTTCTGGG	136
BCL-X TSS +2kb	CCCTGACCCCACCCCAGTTA	TGAGTTTGCCCTGGAACCCC	132
BCL-X TSS -2kb	ACAAGCCGGCCTCAGTTTCC	TCTCTCCCCGGTCCTGATGC	139
BIRC5 TSS	AACCGCCAGATTTGAATCGC	GATGCGGTGGTCCTTGAGAA	108
BIRC5 TSS +2kb	TGAGAATGTTGGAGGGATTTGACA	GCACCTTCTCTCTCCCTGTGG	148
BIRC5 TSS -2kb	CGTCCTTGGTCTGTGCCTGG	GTCCTGCTGATTGGCTCGCT	82
c-MYC TSS	GTCTTTTCTCCCATTCCTGCG	GCCTTCTCCTCTCCCATCTTG	128
c-MYC TSS +2kb	CCACCTCCAGCTTGTACCTGC	AGAAGGCGCTGGAGTCTTGC	135
c-MYC TSS -2kb	TGCGGGTTACATACAGTGCACT	GGAGAGGAGTATTACTTCCGTGCC	150
p57KIP2 TSS	GCTGCCCGCGTTTGC	CGCACTAGTACTGGGAAGGTCC	105
p57KIP2 TSS +2kb	AAAACCGAACGCTGCTCTGC	TAGAGCCCAAAGAGCCCCGA	125
p57KIP2 TSS -2kb	TCGGGATGGGGACTGTCAGA	TCAAAGGCAGGCTGGTTGGG	149
SKP2 TSS	GCGAGCAGCTCTGCAGTTA	TAGCGATATTCGCGGCCTAA	138
SKP2 TSS +2kb	CCACTGCAAAGACCCTGGTG	ACCTCCAGCCTGATCTCCCC	114
SKP2 TSS -2kb	GCTTAGCCTAGGATTCTGCACC	TGCCTCTTGAGGAAGGAATTCA	123

Antibodies			
Target	Cat.-No.	Source	dilution
Rabbit-IgG	sc-2004	SantaCruz Biotechnoloy	1:5000
Mouse-IgG	sc-2005	SantaCruz Biotechnoloy	1:5000
α-tubulin	ab4075	abcam	1:10000
BRD4	ab84776	abcam	1:1000
C-MYC	#13987	CellSignalingTechnology	1:1000
PARP	#9532	CellSignalingTechnology	1:1000
cleaved PARP	#9541	CellSignalingTechnology	1:1000
Caspase-3	#9665	CellSignalingTechnology	1:1000
cleaved Caspase-3	#9664	CellSignalingTechnology	1:500
EZH2	ab3748	abcam	1:1000
p-p57KIP2_T310	#2558	CellSignalingTechnology	1:1000
p57KIP2	#2557	CellSignalingTechnology	1:500
pAKT_S473	#4058	CellSignalingTechnology	1:1000
AKT	#2967	CellSignalingTechnology	1:1000
SKP2	#4358	CellSignalingTechnology	1:500
BCL-Xl	556361	BD	1:2000
Survivin	#2808	CellSignalingTechnology	1:1000
STAT3	#9139	CellSignalingTechnology	1:1000
pSTAT3	#9145	CellSignalingTechnology	1:1000
H3	#3638	CellSignalingTechnology	1:1000
H3Ac	39139	active motif	1:2000
H4Ac	39243	active motif	1:1000
Rabbit IgG isotype control	31887	invitrogen	15µg/ml
H3K27Ac	39133	active motif	15µg/ml
H3K4me3	39915	active motif	15µg/ml
