# Supplementary figures and images for: Combined inhibition of BET proteins and class I HDACs synergistically induces apoptosis in urothelial carcinoma cell lines
Source: Clin Epigenetics. 2018 Jan 4;10:1. doi: 10.1186/s13148-017-0434-3 (PMC5755363; doi:10.1186/s13148-017-0434-3)

## Additional file 2

A

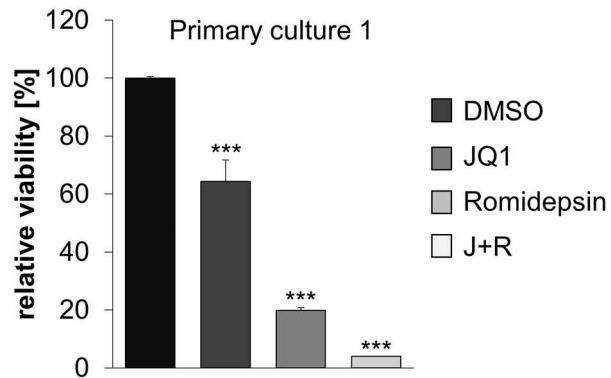

B

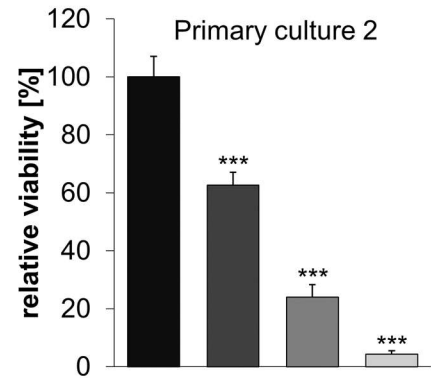

C

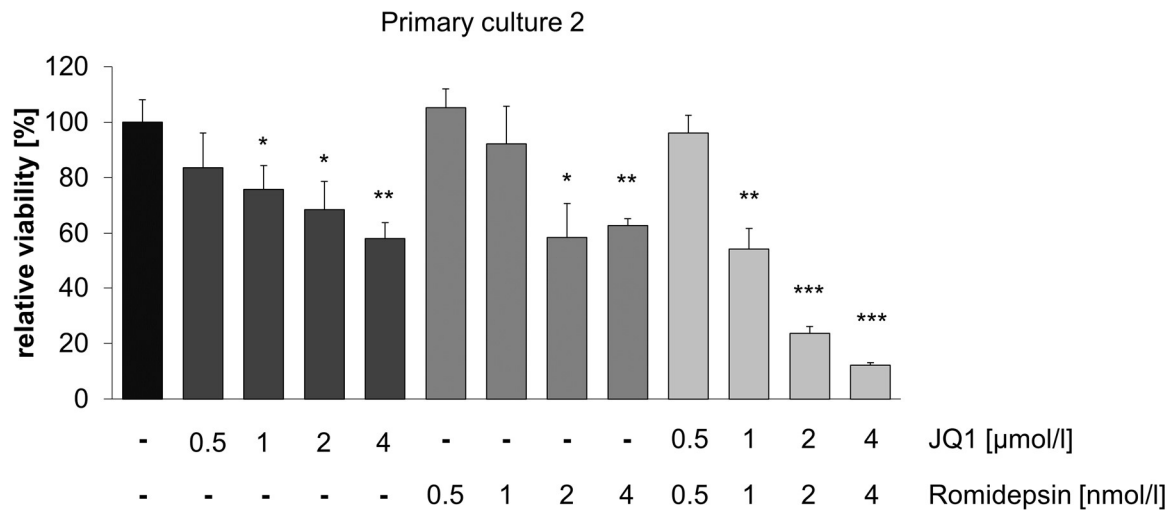

D

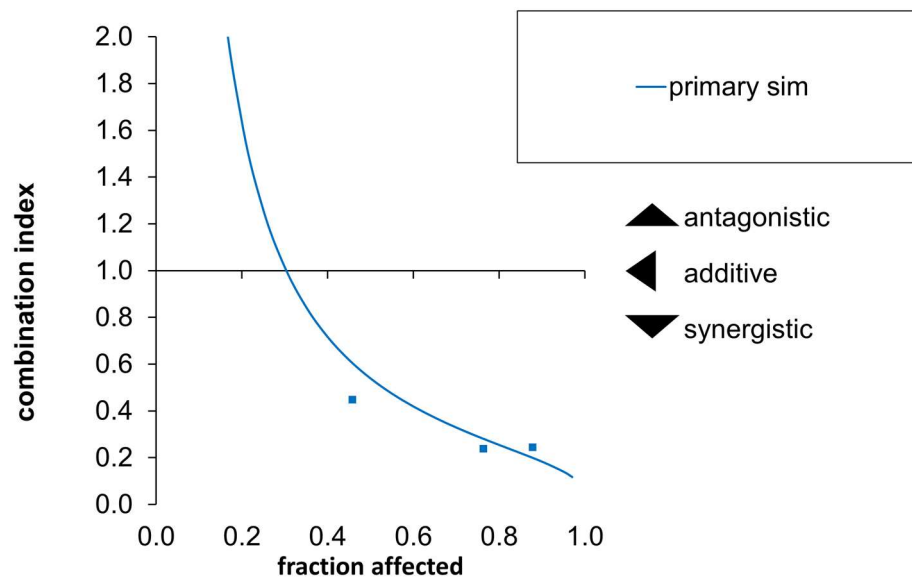

Supplement: Supplementary file 2 — Data on synergistic effects on cell viability of primary cancer cells by combined treatment with Romidepsin and JQ1. (a), (b) Relative viability of primary cultures 1 and 2 established from primary tumor tissue of two different patients after single or combined treatment for 48 h. Relative viability is displayed on the ordinate in percent of the control cells treated with DMSO. (c) Primary culture 2 was used for an extended dose response curve analysis after 24 h to perform Chou-Talalay calculations (***p ≤ 0.001, **p ≤ 0.01, *p ≤ 0.05). (d) Combination index (CI/Fa) plot for the combination of JQ1 and Romidepsin. Cell viability was measured at five constant dose ratio experimental data points by ATP assay for primary culture 2. CI plots were then generated using CompuSyn software. CI < 1 indicates synergism. (PDF 293 kb) [file 13148_2017_434_MOESM2_ESM.pdf]

Additional file 4

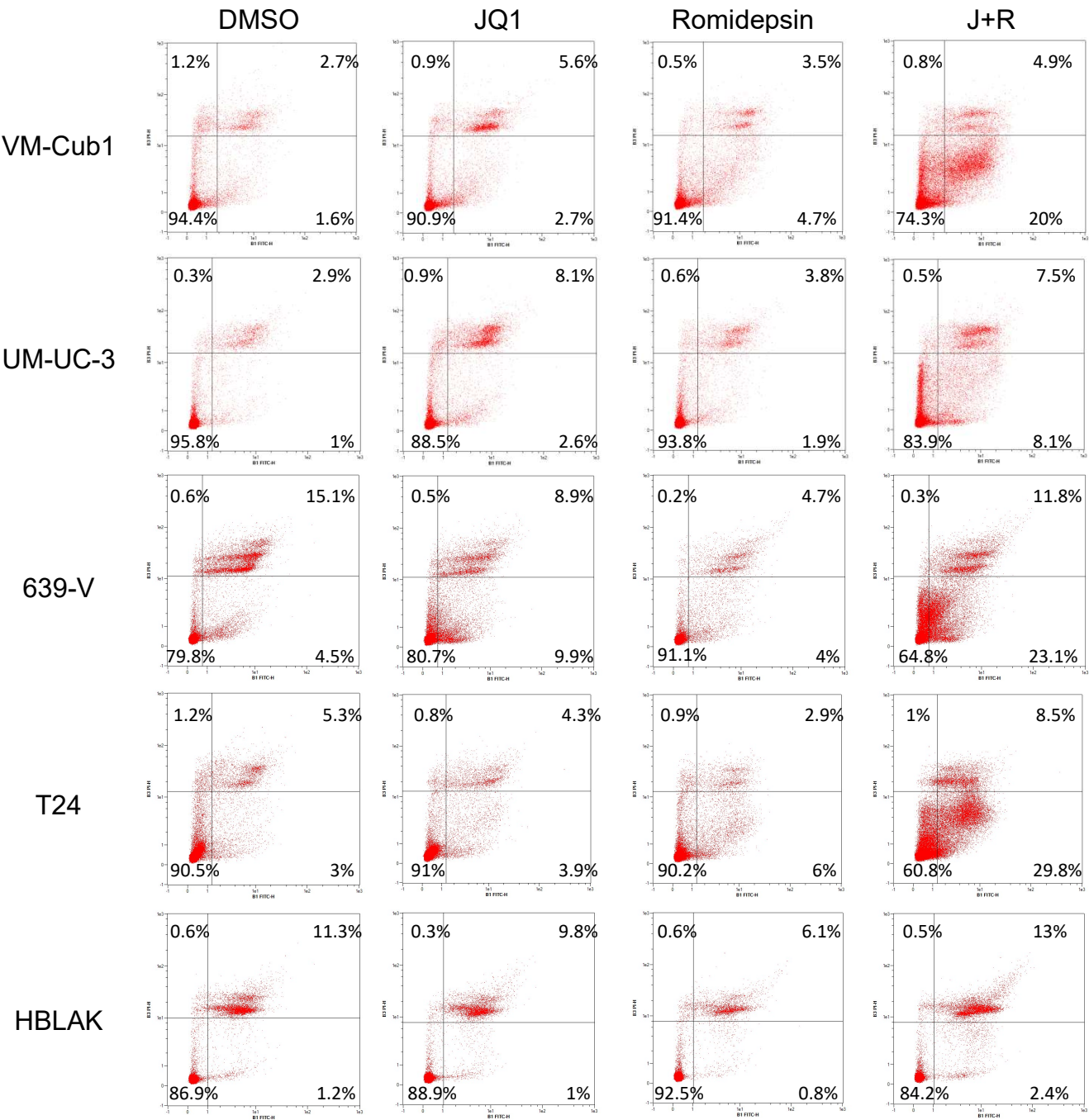

Supplement: Supplementary file 4 — Data on induction of apoptotic cell death by combined treatment with Romidepsin and JQ1. Induction of apoptotic cell death by combined treatment with Romidepsin and JQ1. Flow cytometric analysis of UCCs with indicated treatment after combined staining with PI and Annexin V. Percentages of viable (lower left), early (lower right), or late (upper right) apoptotic and necrotic (upper left) VM-Cub1 cells subsequent to indicated treatments. (PDF 1052 kb) [file 13148_2017_434_MOESM4_ESM.pdf]

## Additional file 5

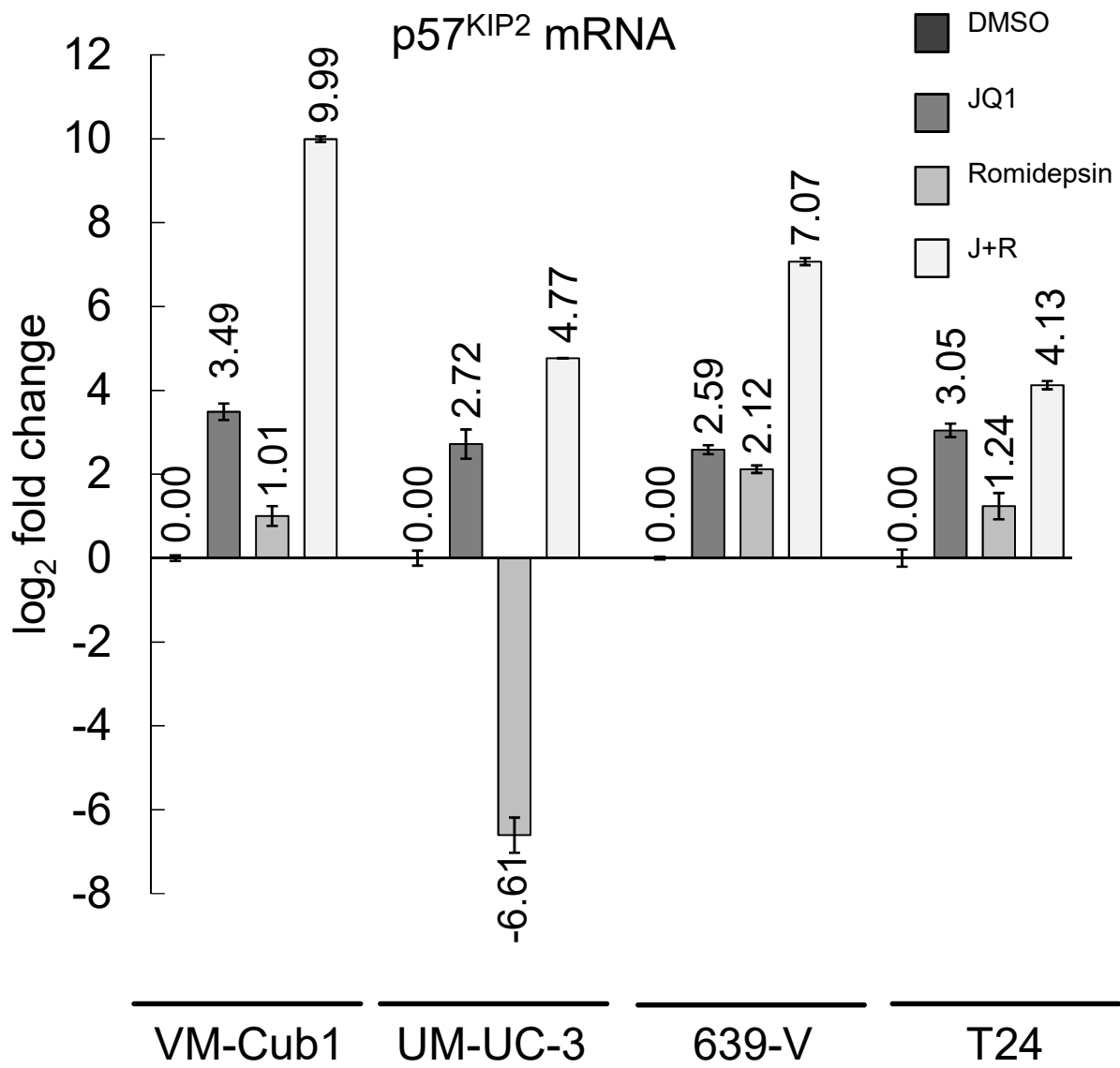

Supplement: Supplementary file 5 — Data on expression changes of of p57KIP2 mRNA by combination treatment in UC cells. Relative mRNA expression levels of p57KIP2 mRNA after single and combined treatment with Romidepsin and JQ1. mRNA levels were measured by qRT-PCR and normalized to the expression of TBP. Fold change compared to DMSO control 48 h after treatment is displayed on the ordinate. (PDF 100 kb) [file 13148_2017_434_MOESM5_ESM.pdf]

# Additional file 6

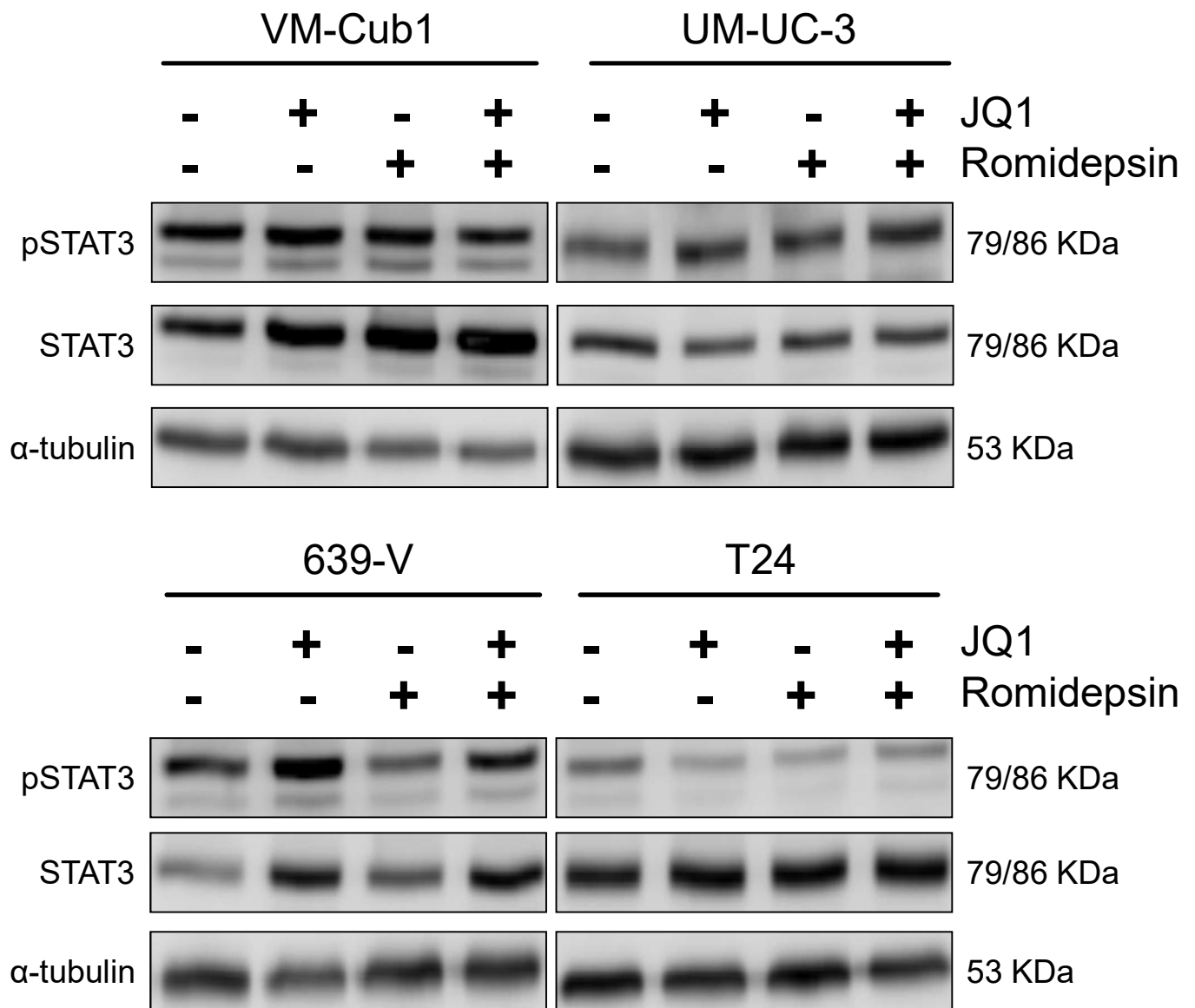

Supplement: Supplementary file 6 — Data on STAT3 activation and expression after combination treatment in UC cells. Phosphorylated and total STAT3 protein was detected by Western blot analysis in four UC cell lines cells after indicated treatment. α-tubulin served as an additional loading control. (PDF 216 kb) [file 13148_2017_434_MOESM6_ESM.pdf]
