# Supplementary material for: Combined inhibition of BET proteins and class I HDACs synergistically induces apoptosis in urothelial carcinoma cell lines
Source: Clin Epigenetics. 2018 Jan 4;10:1. doi: 10.1186/s13148-017-0434-3 (PMC5755363; doi:10.1186/s13148-017-0434-3)
Supplement: Supplementary file 3 — Detailed flow cytometry results from cell cycle analysis. Displayed are percentages of cells in the indicated cell cycle phase as measured by flow cytometric cell cycle analyses following the indicated treatment for 48 h in four different UCCs and in HBLAK cells (Fig. 2d) or in VM-Cub1 knockout cells (Fig. 5d). (PDF 177 kb) [file 13148_2017_434_MOESM3_ESM.pdf]

Additional file 3

Detailed flow cytometry results

To Fig. 2D		sub-G1	G0/G1	S	G2/M [%]
VM-Cub1	DMSO	3.4	64.5	12.4	18.7
	JQ1	3.7	85.5	4.9	5.7
	Romidepsin	3.4	67	2	27.4
	J+R	26.7	23.1	1.9	47.9
UM-UC-3	DMSO	2.3	58.2	22.2	16.4
	JQ1	3.2	54.9	13.8	28.2
	Romidepsin	3.3	55.9	14.7	25.4
	J+R	18.3	23.3	4.4	53.8
639-V	DMSO	0.7	44	20.4	33.7
	JQ1	2.2	50.4	11.1	35.6
	Romidepsin	2.6	47.1	17.1	32.1
	J+R	12.9	48.6	7.7	29.8
T24	DMSO	2.3	54.2	20.4	22.2
	JQ1	1.8	55.8	22.5	19
	Romidepsin	2.5	78.2	5.9	13
	J+R	49.6	37.1	2.4	10.3
HBLAK	DMSO	1	77.6	13.1	7.8
	JQ1	1	86.5	7.5	4.6
	Romidepsin	1.1	84.1	8.1	6.4
	J+R	1.6	95.5	1	1.8

To Fig. 5D		sub-G1	G0/G1	S	G2/M [%]
VM-Cub1 parental	DMSO	1.5	58.8	17.8	21.1
	J+R	16.7	37.4	3.4	42
VM-Cub1 p57$^{(-/-)}$	DMSO	5.2	49.7	21.4	22.8
	J+R	67.1	8.5	9.7	14.2
